# Sensorized Tip for Monitoring People with Multiple Sclerosis that Require Assistive Devices for Walking

**DOI:** 10.3390/s20154329

**Published:** 2020-08-03

**Authors:** Asier Brull, Asier Zubizarreta, Itziar Cabanes, Ana Rodriguez-Larrad

**Affiliations:** 1 Department of Automatic and Control Systems, University of the Basque Country UPV/EHU, Faculty of Engineering of Bilbao, Ingeniero Torres Quevedo Square, 48013 Bilbao, Biscay, Spain; asier.brull@ehu.eus (A.B.); itziar.cabanes@ehu.eus (I.C.); 2 Department of Physiology, University of the Basque Country UPV/EHU, Faculty of Medicine and Nursing, Barrio Sarriena, s/n, 48940 Leioa, Biscay, Spain; ana.rodriguez@ehu.eus

**Keywords:** sensorized tip, patient monitoring, multiple sclerosis, wearable sensors, rehabilitation

## Abstract

Multiple Sclerosis (MS) is a neurological degenerative disease with high impact on our society. In order to mitigate its effects, proper rehabilitation therapy is mandatory, in which individualisation is a key factor. Technological solutions can provide the information required for this purpose, by monitoring patients and extracting relevant indicators. In this work, a novel Sensorized Tip is proposed for monitoring People with Multiple Sclerosis (PwMS) that require Assistive Devices for Walking (ADW) such as canes or crutches. The developed Sensorized Tip can be adapted to the personal ADW of each patient to reduce its impact, and provides sensor data while naturally walking in the everyday activities. This data that can be processed to obtain relevant indicators that helps assessing the status of the patient. Different from other approaches, a full validation of the proposed processing algorithms is carried out in this work, and a preliminary study-case is carried out with PwMS considering a set of indicators obtained from the Sensorized Tip’s processed data. Results of the preliminary study-case demonstrate the potential of the device to monitor and characterise patient status.

## 1. Introduction

It is estimated that 2.3 million people in the world suffer from Multiple Sclerosis (MS) [1]. This neurological disease has an important impact on society, as it is a chronic degenerating disease with no cure that appears in young people [2]. This implies not only an important economic impact on the health system, but also in the active population, as the physical decline causes inability to work at early ages [3].

MS is an autoimmune disease that affects the Central Nervous System, more specifically the brain and spinal cord. Although its symptoms vary depending on the area affected by the disease, some of the most common ones are fatigue and motor dysfunction. After 15 years from diagnosis, most of the patients require Assistive Devices for Walking (ADW) such as crutches or canes to maintain their autonomy [4]. This work focuses on providing solutions for the needs of these patients.

At present there is no cure for MS. Hence, the treatment focuses on the prevention of new attacks and alleviating the symptoms [5]. In this task, rehabilitation plays a key role, as it has demonstrated to be effective for reducing the effect of the symptoms caused by the disease [6,7], and therefore being central for maintaining the highest possible level of independence as the disease progresses. However, in order to be effective, therapies have to be adapted to each patient [8], which is not a trivial task.

Technological solutions can provide the information required for this purpose, by monitoring patients and extracting relevant indicators to assess their status. Some of the most popular devices for this purpose are *wearable sensors*, which are based on inertial sensors (accelerometers, gyroscopes) that allow to monitor limb motion [9,10,11,12]. For this purpose, wearable sensors are usually attached to the body of the patient. Although these devices are widely used in a range of applications, this latter requirement may generate rejection on PwMS that require ADW, as they have difficulties to move and are very sensitive to any external attachment to their limbs.

Hence, in order to provide a contactless monitoring approach some works have proposed the use of the internal sensors of smartphones to capture the motion of the lower limb once placed in a pocket of the trousers [13,14]. However, the data processing is more complex, as the mobile phone is not fixed and parasitic motions arise.

To overcome these aforementioned issues, recent studies [15] have proposed to include sensors in passive, daily-use Assistive Devices for Walking, such as canes or crutches, as they can provide more reliable monitoring data. Most of the works in this area [16,17,18,19,20,21,22,23,24] propose devices capable of measuring the load applied, as well as their motion, which can be used to define indicators that could provide relevant information regarding the modality of use of the ADW. To better characterize this use some works also include sensors that measure the force applied to the handle [25] or sensors to detect nearby objects [26].

The measurement of the applied axial load, is typically carried out considering a force sensor [17,24,26,27], which is usually expensive but provides an accurate measurement. Other authors propose the use of strain gauges [16,18,19,20,21,22,23,25], whose cost is lower, as well as their accuracy. The motion of the device is traditionally measured by the use of IMUs (Inertial Motion Units), which include accelerometers and gyroscopes. These devices, however, do not provide directly the orientation of the crutch or cane, and different techniques have to be applied to estimate them. One of the most simple approaches is based on the direct integration of the gyroscope rotational speed, and the periodical correction of the accumulated errors [19]. However, most authors use either a quasi-static approach, determining the orientation from the projection of the gravity vector on each acceleration axis [16,18,25,26]; or different filters such as Kalman [17,28,29] or CAHRS [30]. The aforementioned data is captured by an on-board Data Adquisition Device (DAQ), which is typically self-designed [18,23,26,27,31], although there are approaches based on Arduino or rapid-prototyping devices [21].

The raw data captured by the sensors of the device, however, is not generally useful for the therapist without further processing. Hence, in the literature, different indicators based on the raw data are defined. This way a change in these can be used to detect changes in the status of the patient. In the case of ADW, most of the indicators are related to the axial force, such as the maximum [32,33] or average load [33] with respect to the weight of the patient. For people that do not require these devices, on the other hand, the number of steps required for a given distance, the average speed or the time between steps [34] are some of the most used.

As previously analysed, currently there are a number of works that have proposed instrumented crutches and canes, with different sensor solutions and indicators extracted. However, this is still an open area, where further research is required—first, only a few of the proposed devices have been validated, characterising their measurement error [17,20,23,24,26,27]; second, only some of the works evaluate (preliminary) the proposed device with patients, defining indicators from the sensor data and demonstrating some kind of correlation between the proposed indicators and the patient status [17,21,24,26]; third, due to the complexity of the device orientation estimation, most of the indicators proposed are related with the applied axial load, although the former variables could be used to characterise the pattern of use of the sensorized ADW; and finally, all the aforementioned monitoring systems are part of a generic assistive device, and cannot be adapted to the personal ADW of each patient, which is usually personalized to his/her needs.

Hence, in this work a novel monitoring device is proposed, with four main contributions—(1) the proposed device is a universal Sensorized Tip that can be fit to the patient’s own personalised ADW, which minimizes the impact of the solution and consequently, improves its acceptability; (2) a complete and thorough validation of the device’s monitoring capabilities is presented, detailing the algorithms used to estimate relevant parameters and validating them experimentally; (3) a novel procedure to estimate relevant orientation angles for the tip with respect to the body motion is proposed; and (4) the patient status assessing capabilities of the device are demonstrated by carrying out a preliminary/exploratory use-case study with PwMS. Note however, that the goal of this latter contribution is not to perform a thorough correlation analysis, but to demonstrate the potential of the device.

The rest of the work is structured as follows—Section 2 details the proposed Sensorized Tip and its capabilities; Section 3 characterises the measurement errors of the proposed device; Section 4 details the procedure to estimate the angles of motion of the Sensorized Tip; Section 5 illustrates the use of both raw sensor and estimated orientation data to define a set of indicators to perform a preliminary analysis of the capabilities of the device to assess PwMS status in a exploratory use-case study; finally, the most important ideas are summarised in Section 6.

## 2. Sensorized Tip Prototype

**Figure 1:**
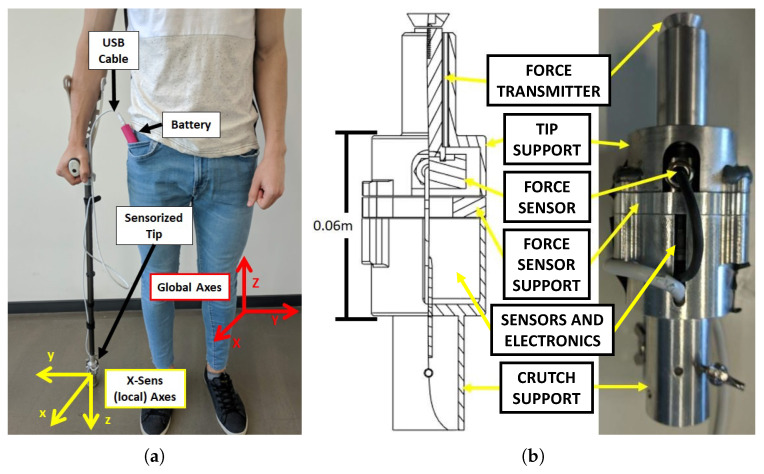
(**a**) System elements and reference axes. (**b**) Sensorized Tip mechanical structure.

The general structure of the developed Sensorized Tip is detailed in Figure 1a. The overall system is composed by two main elements: the Sensorized Tip, whose structure is made in aluminium and integrates the sensors and the Data Acquisition (DAQ) device; and an external standard USB based 5 V battery. The Tip is designed to be attached in any crutch or cane to monitor its motion by means of a couple of screws. Moreover, it is designed so that it can be easily repaired if damaged. The weight of the Sensorized Tip prototype, without taking into account the external battery that is attached, is 180 g.

The Sensorized Tip integrates three sensors in its structure: a force sensor, a barometer and an Inertial Motion Tracking Module, whose specifications will be detailed in Section 2.1. The data provided by these sensors is processed by a low cost B;uetooth Low Energy (BLE) Nano v2 board based on the nRF52 IC processor, which is able to communicate with Bluetooth 4.0 Low Energy (BLE) protocol with an external acquisition system, such as a PC or a mobile phone.

The battery is based on a 5 V 2600 mAh power bank typically used to charge mobile phones with a mass of 75 g. The use of standard USB-A based connectors was selected to simplify the design and increase compatibility. The decision to put the battery outside the Tip is motivated by the need to reduce the mass of the Tip and minimize its impact on the patient. Note that PwMS usually present progressive physical deterioration and high risk of falling, and incorporating even a small mass into the ADW can notably difficult their mobility. The cable connecting the sensor tip to the battery is attached to the crutch or cane by means of straps.

Next, each of the elements composing the Sensorized Tip are detailed.

### 2.1. Integrated Sensors

As analysed in Section 1, the most important basic variables that define the use of crutches or canes are the motion (acceleration, speed, angles, altitude) and the overall force applied on the walking support device. Based on this, three sensors have been selected.

The MTi-1 by X-Sense is an Inertial Measuring Unit (IMU) that integrates a 3-axis accelerometer (range ±156.96 m/s${}^{2}$), gyroscope (range ±2000 ${}^{\circ }/s$) and magnetometer (range ±0.8 *G*) with an algorithm that provides Attitude and Heading Reference System (AHRS) functionalities. Hence in addition to the linear acceleration and rotational speed with respect to the local axes (Figure 1a), it provides the absolute Roll-Pitch-Yaw angles of the Tip in a Global Reference Frame with a relative low error (Roll and Pitch dynamic error of 0.5°, and Yaw dynamic error 1°), providing a 3D representation of the Tip orientation in real-time.

For the measurement of the force applied to the longitudinal axis of the crutch, a C9C force sensor manufactured by HBM is used. This element is designed to measure static and dynamic compression forces of up to 1 kN (0.2% precision). Due to the mechanical disposition of the sensor (see Section 2.2) a preload is required to ensure that low forces are measured. In addition, as the sensor is of piezoelectric nature, a conditioning circuit based on a INA118 amplifier is used to amplify the generated voltage to a suitable level.

Finally, the measurement of altitude is carried out using a low cost BMP280 sensor manufactured by Bosch, which communicates using I2C protocol with the Sensorized Tip’s processing unit. This sensor allows to determine if the patient is going up or down stairs or slopes.

Although these sensors are commercial, a calibration procedure has been carried out in Section 3 in order to quantify the measurement error once implemented in the Sensorized Tip.

### 2.2. Mechanical Structure

The Sensorized Tip’s structure has been self-designed to integrate the aforementioned sensors and the BLE Nano processing unit. The structure has been manufactured in aluminium to reduce its weight. In addition, its longitudinal size (0.06m) has been selected to minimize the need of adjusting the crutch height, as 0.06m are equal to three discrete positions in the length adaptability of a standard crutch.

The elements composing the structure and their disposition are detailed in Figure 1b. The Data Processing Unit and the required electronics are placed in the upper part of the Tip, housed by an aluminium enclosure (Crutch support). The force sensor is placed in the lower part, attached to a stiff aluminium support (Force sensor support). The axial force applied on the Tip is transmitted by the Force transmitter, which is a mobile part which can transmit force axially. The rubber Tip of the crutch or cane is attached to the Tip support. Note that this element is dependant on the particular one selected by the patient for his/her needs.

As stated before, a preload is required to ensure proper operation of the force sensor. For that purpose, the set of screws that join the Crutch Support, Force Sensor Support and Tip Support parts are tightened to a desired preload and fixed with nordlock washers.

### 2.3. Data Processing Unit

The selected data processing unit is a BLE Nano board, which is based on the nRF52 microcontroller. This integrated circuit provides Data Acquisition (DAQ) capabilities and Bluetooth 4.0 Low Energy (BLE) connectivity with a very low energy consumption, which allows the system to work for a whole day with a small battery.

**Figure 2:**
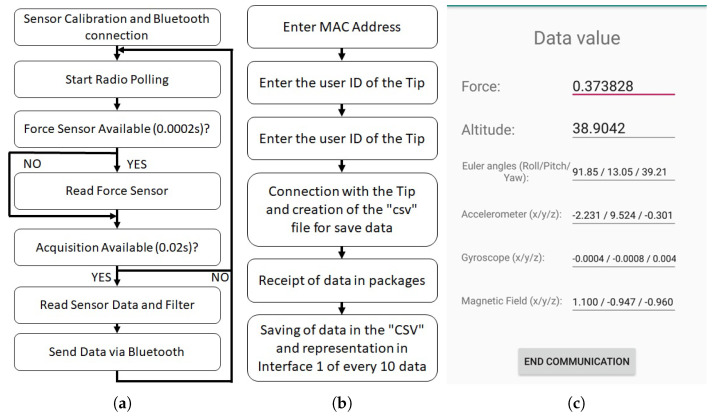
(**a**) Operation scheme of the Bluetooth Low Energy (BLE) Nano V2 software, responsible for capturing the data coming from the sensors and sending them via Bluetooth. (**b**) Scheme of operation of the PC/Mobile Phone interface. (**c**) Representation of one of the screens of the smartphone application developed for the storage of the data received from the Sensorized Tip.

The tasks performed by the BLE Nano are summarised in Figure 2a. After the initialisation of the Bluetooth connection and the calibration of the sensors, it performs several tasks periodically: first, it captures the data from the aforementioned sensors; second, it compacts the data and sends it using BLE protocol with 50Hz rate to an external logging device (PC or mobile phone). Note that this frequency is enough to capture the walking data with assistive devices [35]. The capture process is analysed next.

The sensor data is captured using two different approaches depending on the nature of the sensor output. This way, both the barometer and the MTi-1 module are connected using I2C communication protocol, hence, their measurements are requested each 20ms by the processing unit and stored in memory prior to sending them. The force sensor, on the other hand, is read using an analog input. As the measurement of the force sensor can be noisy, the force sensor is measured at 5 kHz rate, and its data is filtered prior to storing its value in memory.

The sensor data stored internally in the processing unit memory is sent periodically using Bluetooth 4.0 Low Energy to a logging device. The Attribute Protocol (ATT) is used so that once the Sensorized Tip and the logging device have been paired, communication is carried between both devices. This way, each 20ms (50Hz), 38 bytes of sensor data are sent in two packets (20 bytes each). Package data is summarized in Table 1.

Note that the Sensorized Tip is designed so that an external logging device is required to store the data. For that purpose, any device that supports BLE protocol can be used. The developed prototype provides a PC based logging system and an Android-based mobile phone app (Figure 2c) to perform these tasks. The logging programs developed in both devices follow the steps defined in Figure 2b. As it can be seen, the data is stored in a csv file. In order to detect possible package losses in the wireless transfer, the *iteration* bits are used to identify them (Table 1).

## 3. Characterization of the Measurement Errors

The aim of this section is to characterize the measurement errors associated to each of the three integrated sensors: the Inertial Motion Processing Unit (velocities, accelerations and angles), the barometer and the force sensor. This will allow to validate the accuracy of the proposed Sensorized Tip to measure crutch motion.

In order to achieve this goal, a series of calibration and measurement tests have been carried out with the Sensorized Tip at the research facilities of the University of the Basque Country (UPV/EHU). In particular, the high-fidelity measuring equipment of both the Automatic Control Laboratory and the 3D Body Motion Capture Laboratory were used to evaluate the measurements provided by the Sensorized Tip.

Next, each test is described, detailing the used equipment to characterize the measurement errors associated to each data source of the Sensorized Tip.

### 3.1. Euler Angles

The Mti-1 sensor from X-Sens integrated in the Sensorized Tip has an internal algorithm that provides an estimation of its orientation in a global reference frame (see Figure 1a). Using the internally estimated Euler angles, the real-time orientation of the crutch in 3D space can be estimated, as it will be detailed in Section 4.

In order to validate the estimations provided by the Mti-1 sensor, a series of tests were carried out comparing these with the data provided by the VICON 3D Motion Capture System installed in the 3D Motion Capture Laboratory. This facility provides a wide empty area with a flat soil where eight high fidelity Vicon Motion Capture System (MCS) cameras and a high precision Bertec 4060-15 force platform can be placed to capture accurately 3D motion data in a predefined working area.

**Figure 3:**
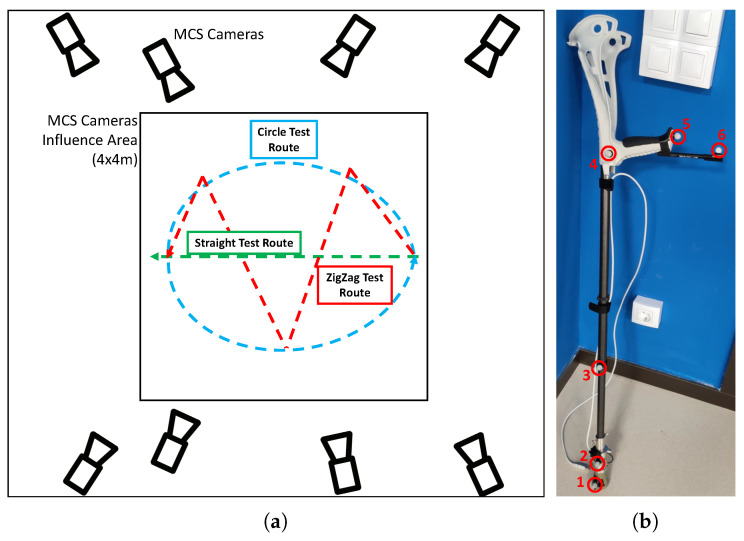
(**a**) 3D Motion Capture Laboratory Schematic with camera placements, capture area and defined test trajectories. (**b**) Reflective marker distribution on the tested crutch (the markers are identified with numbers).

For the error characterization measurements defined in this section, the eight cameras were placed to cover an area of 4x4m in the center of the lab, which was free of obstacles as seen in Figure 3a. To capture the motion of the crutch, and extract its 3D motion, 6 reflective markers were placed in a standard crutch (Figure 3b). Note that as the global reference system of both the Mti-1 sensor and the MCS were not the same, a calibration procedure was first executed to calculate the appropriate transformation matrices that relate both reference systems. In addition, in order to synchronize the data captured by both devices (Sensorized Tip and MCS), an initial vertical blow over the MCS force plate was executed to indicate the start of each measurement.

**Figure 4:**
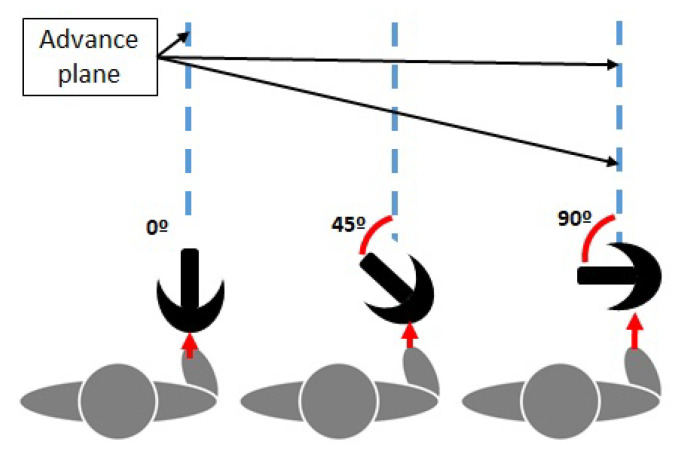
Position of the crutch handle with respect to the advance plane, in the different validation tests of Euler angles provided by the X-Sens.

In order to characterize the measuring error of the Sensorized Tip, the way patients use ADW was considered. For instance, depending on the ADW, patients tend to grab its handle with an angle with respect to the advance plane (Figure 4). This way, to analyze the measurement errors in different scenarios, five trajectories were considered: three 4m straight walking tests, with different crutch handle orientations (more or less 0°, 45° and 90° rotation), a zig-zag trajectory (0° rotation) and a circular trajectory (0° rotation). These trajectories were marked in the floor as reference, as seen in Figure 3a.

Two members of the research group executed the tests, executing each trajectory twice at a normal pace (always in the same direction), once the required synchronizing initial vertical blow over the MCS force plate was executed. Data from the researchers is provided in Table 2.

**Figure 5:**
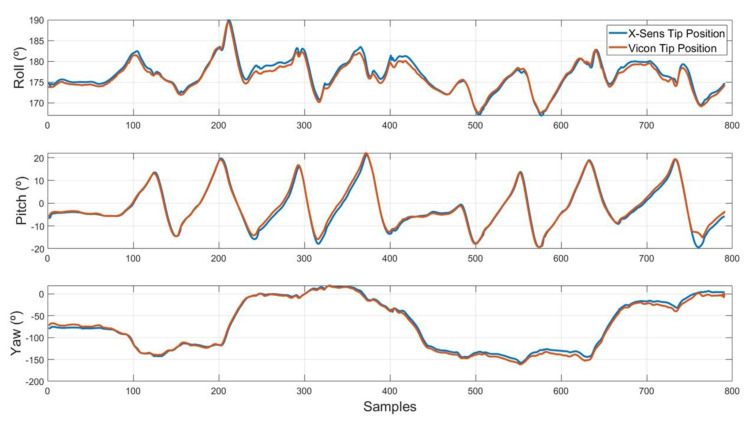
Euler angles of Vicon Motion Capture System (MCS) and X-Sens comparison in zigzag test.

Results of the measurement RMS (Root Mean Square) error in the MTi-1 reference system are summarized in Table 3. It can be seen that the Sensorized Tip provides a reduced estimation error for the Roll and Pitch angles (below 1.5 degrees RMS), while the error is slightly higher in the Yaw angle (up to 4.3 degrees RMS). The dynamic error specifications are higher than those specified by the manufacturer (0.5° for pitch/roll and 2° for yaw), although similar to other approaches proposed in the literature and acceptable for the required application. This can also be seen in Figure 5, which shows the time evolution of roll/pitch/yaw angles for both the Mti-1 and Vicon MCS measurements for the zig-zag trajectory experiments.

### 3.2. Gyroscope

The Mti-1 by X-Sens integrates a gyroscope and accelerometer, whose raw data can also be captured. In this section, the gyroscope measurements will be analyzed and the measurement errors characterised. It is to be noted that the Mti-1 internally performs a calibration each time it is activated. However, it is widely known gyroscopes present a drift with time, which X-Sens ensures it is below 10 ${}^{\circ }/h$ and will not be analyzed in this section.

In order to determine the angular speed measurement error, an AKD21C servomotor configured in speed mode with a high precision encoder was used. The Sensorized Tip was attached to the axis of the servomotor using a 3D printed base, which allows to position the device so that each of the three local axes of the Mti-1 sensor (*x*, *y*, *z*) (Figure 1a) are aligned with the rotation axis of the motor. Each axis measurement is tested at three different constant angular speeds: 100 ${}^{\circ }/s$, 200 ${}^{\circ }/s$ and 300 ${}^{\circ }/s$. These speeds were verified with an external tachometer at the Automatic Control Laboratory.

Results are detailed in Table 4. As it can be seen, errors are low at 100 ${}^{\circ }/s$, increasing with higher speeds, with the exception of the speed of 300 ${}^{\circ }/s$ in the z axis in which the error decreases. In general, error in *z* axis is higher than in the rest due to slight misalignment in the location of the MTi-1 sensor with respect to the center of the Sensorized Tip.

However, it has been experimentally checked that an average healthy person generates angular speeds on the walking assist system less than 180 ${}^{\circ }/s$ in *x* and *y* axes and 220 ${}^{\circ }/s$ in the *z* axis. Hence, an error less than 1 ${}^{\circ }/s$ is guaranteed in this range, which is acceptable for this application.

### 3.3. Accelerometer

In order to characterize the accelerometer data provided by the Mti-1, first the calibration procedure proposed in Reference [28], which uses the gravity vector as a reference, is applied, and the accelerations in the global reference frame are calculated. Then, the data provided by the accelerometer is compared with the acceleration data obtained by the Vicon MCS system.

**Figure 6:**
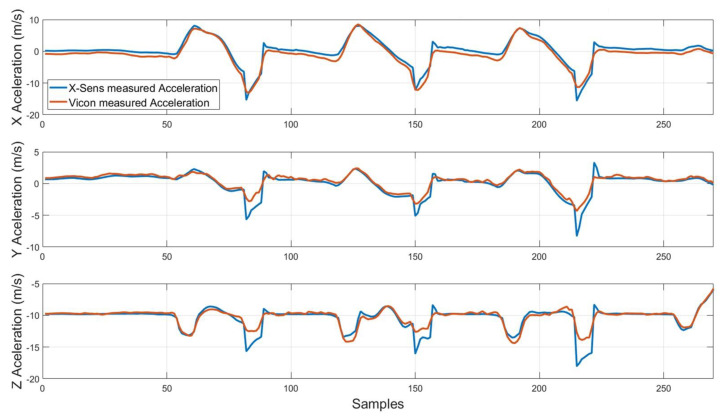
Comparison of acceleration measured by X-Sens and Vicon.

The 4 meter walking test (0${}^{\circ }$ rotation) dataset defined in the Section 3.1 is used to perform this characterization, whose time evolution is depicted in Figure 6. Note that the captured accelerations are first filtered using a low pass filter.

Table 5 summarizes the RMS and average errors in the global reference frame for each axis. As it can be seen, the RMS estimation error is generally less than 1.4 m/s${}^{2}$, which is acceptable for the monitoring application. In addition, note that, as seen in Figure 6, the acceleration measurements along the experiment have good quality, except when the Sensorized Tip impacts the ground, in which higher accelerations appear due to this effect.

### 3.4. Force Sensor

In order to characterize the force sensor measurement, its measurements were compared with the ones given by the Bertec 4060-15 force plate integrated in the 3D Motion Capture Laboratory, as previously detailed.

It is important to note that, due to the Sensorized Tip design, the force sensor measures the force transmitted by the rubber Tip to the *force transmitter* part (Figure 1b). Hence, friction and damping effects appear, requiring to calibrate the force sensor prior to its use. For that purpose, the Sensorized Tip was placed vertically over the force plate using a 3D printed support, and a set of constant loads in the 0–100 kg (approximately 0–1 kN) range were progressively applied to it. Using the measurements of both the force plate and the Sensorized Tip force sensor the following calibration curve was obtained,

(1) $$y=-230.71x^{2}+797.3736x-285.6619.$$

**Figure 7:**
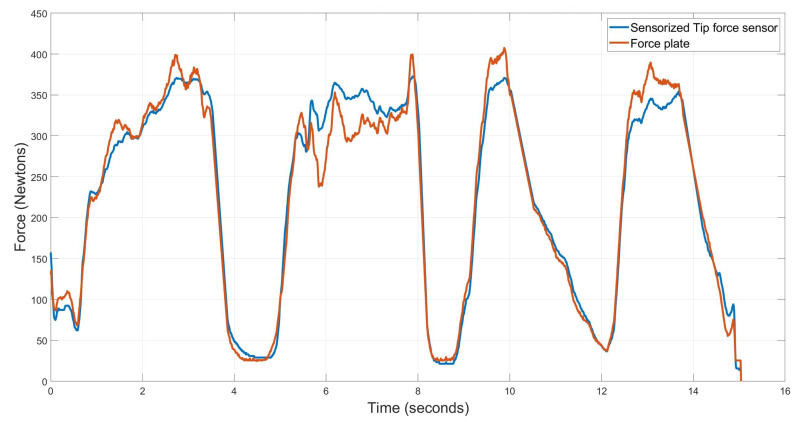
Force sensor after calibrate curve and data obtained from the scale.

Once calibrated, the Sensorized Tip was attached to a crutch and different loads were applied after placing it on the Bertec 4060-15 force plate. A particular test is shown in Figure 7. As it can be seen the negative and positive force gradients match, but there exist a measurement error when the maximum force is applied to the crutch due to the force transmission mechanism of the design and the existing friction. An average error of –0.0425 N is obtained from this sensor, with an RMS of 21.1104 N, which is considered acceptable for this application.

### 3.5. Barometric Sensor

Finally, the Sensorized Tip includes a BMP280 barometer which allows to calculate the relative height based on the atmospheric pressure. In order to determine the measurement accuracy of the sensor, a simple test was carried out, consisting on using a stair set as a reference. It was divided into four flights, with 12 stairs per flight and a total of 2.04 m between flights.

**Figure 8:**
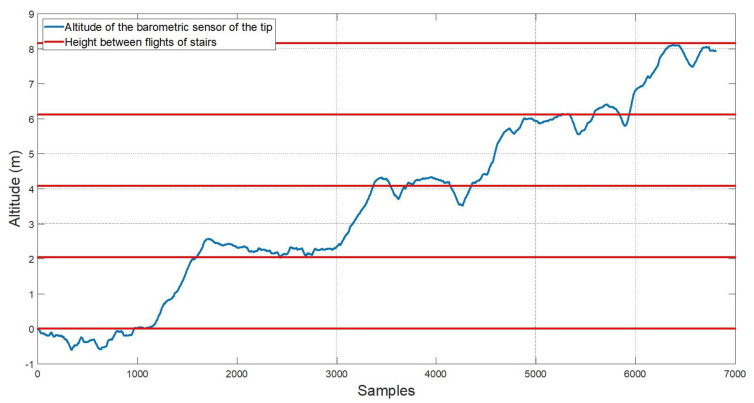
Comparison and validation of the altitude provided by the barometric sensor and the real altitude.

Results are shown in Figure 8, in which the starting floor was considered as the 0 height value. If the *flat* areas associated to each flight of stairs are considered in the time evolution, the RMS error is 0.2716 m, while the average error is –0.0466 m. Note that this fits the data from the manufacturer, which offers a relative precision of 0.12 hPa.

## 4. Estimation of the Orientation of the Device

As detailed in the introduction, orientation of the ADW can be used to define indicators related to the patterns of use of the device, which are related to the status of the patient.

However, the measurement of the orientation is not a trivial task, and as analysed previously, different approaches exist. In the particular case of the proposed device, the integrated Mti-1 sensor provides, using a proprietary algorithm based on a Kalman Filter, the Euler orientation angles that relate the local Sensorized Tip reference system $S_{tip}\left(x,y,z\right)$ with a global reference system $S_{G}\left(X,Y,Z\right)$.

**Figure 9:**
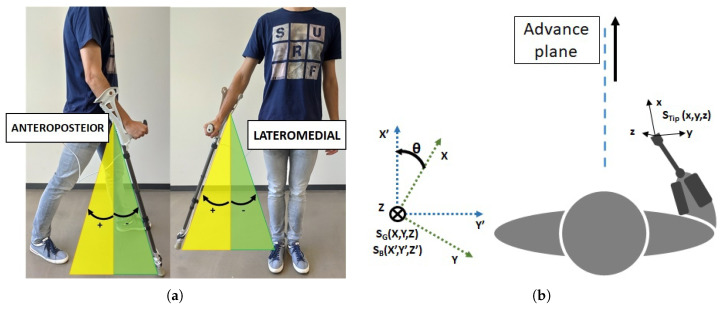
(**a**) Anteroposterior and Lateromedial angles. (**b**) Global, Body and Sensorized Tip reference frames.

However, for the specific application of the Sensorized Tip, the relative motion of the assistive device with respect to the body of the patient is required. This is, the lateromedial and anteroposterior angles, as seen in Figure 9a.

The calculation of these angles is not trivial and a two step procedure has been defined to estimate them. First, a *body reference system* ($S_{B}\left(X^{\prime },Y^{\prime },Z^{\prime }\right)$) has to be inferred from the data provided by the Sensorized Tip (Figure 9b). Second, the calculation of the lateromedial and anteroposterior angles is carried out by projecting the Sensorized Tip reference system into what will be named *advance plane*, that is, the $X^{\prime }Z^{\prime }$ plane of the body reference system, related to the direction of movement of the patient. This procedure will be detailed next.

### 4.1. Estimation Algorithm for the Body Reference System and the Advance Plane

The *body reference system* is considered aligned with the *Z* axis of the global reference system of the Mti-1, but its $X^{\prime }$ axis always points in the direction of the body motion, which defines the advance plane $X^{\prime }Z^{\prime }$. Hence, as seen in Figure 9b, the global and body reference systems are related by rotation of θ with respect to the global *Z* axis.

Hence, in order to define the *body reference system*, the motion direction in the (XY) plane of the global reference system has to be defined. Note that this is not a trivial tasks as—(1) the global reference system of the Mti-1 only ensures the *Z* axis, but the *X* depends on an internal magnetometer which is affected by electromagnetic noise; and (2) as seen in Section 3.1, patients may grab the handle of the ADW with multiple angles, or even place the Sensorized Tip misaligned with the crutch.

**Figure 10:**
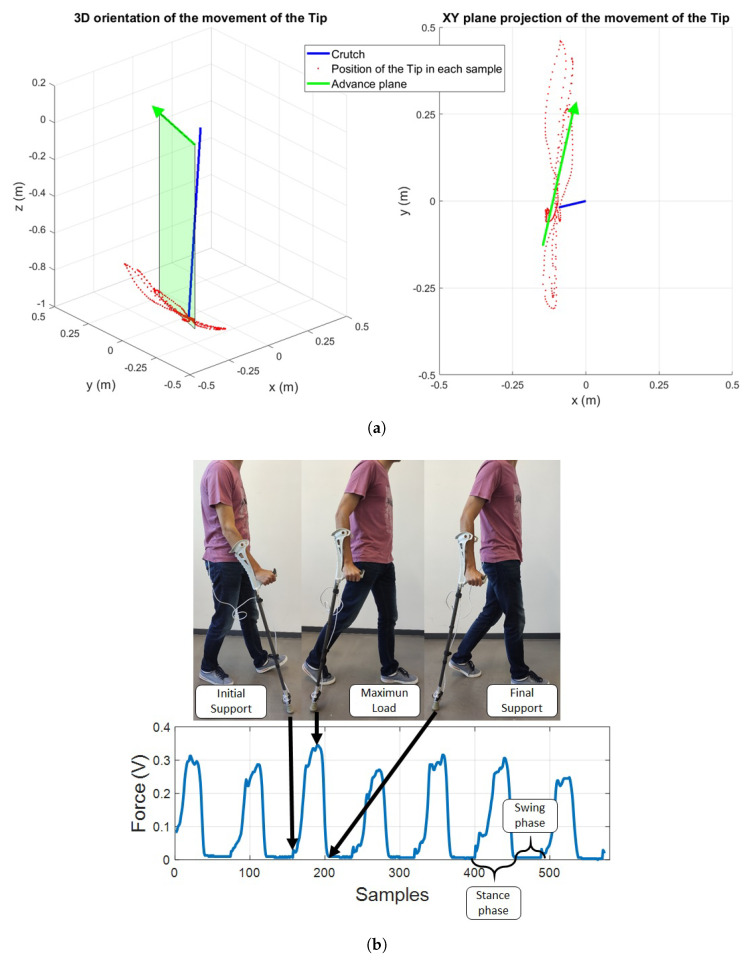
(**a**) 3D Orientation of the Sensorized Tip and (XY) plane projection. (**b**) Stance and Swing Phases.

In this work a novel approach is proposed to estimate the motion direction, and thus, the advance plane and body reference system, based on the *predominant* direction defined by the projection of the Sensorized Tip motion in the XY plane. The basic idea is to project on this plane the vector defined by the Euler angles provided by the Mti-1, creating a set of points in which a linear regression is applied to detect the main direction (Figure 10a).

The proposed procedure is applied to each ADW cycle (see Figure 10b). When using a crutch or cane, in each cycle two phases can be differentiated if the load force is considered—the stance phase, in which the patient applies load to the assistive device; and the swing phase, in which no contact with the ground exists and the device is moved through the air to the next stance phase start.

This way, when the Sensorized Tip detects that the stance phase has started, the absolute orientation data (Euler angles) is captured within this phase. When the stance phase ends, the captured Euler Angles (α, β, γ) are used to represent in 3D the orientation of the ADW. For each set of Euler angles captured, the following rotation matrix can be defined,

(2) $${}^{G}R_{tip}=R_{z}\left(\alpha \right)\;R_{y}\left(\beta \right)\;R_{y}\left(\gamma \right)=R_{\mathrm{rpy}},$$

which relates the Sensorized Tip local reference system and the global reference system of the Mti-1. As the Sensorized Tip local *Z* axis is aligned with the Sensorized Tip / assistive device axis, it is possible to define the representation of unitary vector ${}^{G}u_{z}$ associated to the local *Z* axis in the global reference frame by extracting the third column of $R_{\mathrm{rpy}}$,

(3) $$R_{\mathrm{rpy}}=\left[\begin{array}{ccc} {}^{G}u_{x} & {}^{G}u_{y} & {}^{G}u_{z}. \end{array}\right]$$

The projection of ${}^{G}u_{z}$ in the XY plane of the Global Reference System reflects the motion of the Sensorized Tip in this reference system. Hence, if a particular stance phase is evaluated, cloud of points is obtained, which reflects the motions carried out in that particular cycle (Figure 10a).

Considering that no turns are carried out within an stance phase, a linear regression is applied to the projected points in the XY plane, obtaining the *predominant* direction of the body in that stance phase. Thus, the angle of this line with respect to the global reference system *X* axis defines θ (Figure 9b), and the *body reference system* can be calculated as,

(4) $${}^{G}R_{B}=R_{z}\left(\theta \right).$$

In order to validate this approach, the data of the tests carried out in Section 3.1 are used, in which five different trajectories were tested: 4m walking straight with different crutch handle orientations (0${}^{\circ }$, 45${}^{\circ }$ and 90${}^{\circ }$), zig-zag trajectory and circular trajectory. The data from the Vicon MCS system was used to evaluate both body and crutch motions.

**Figure 11:**
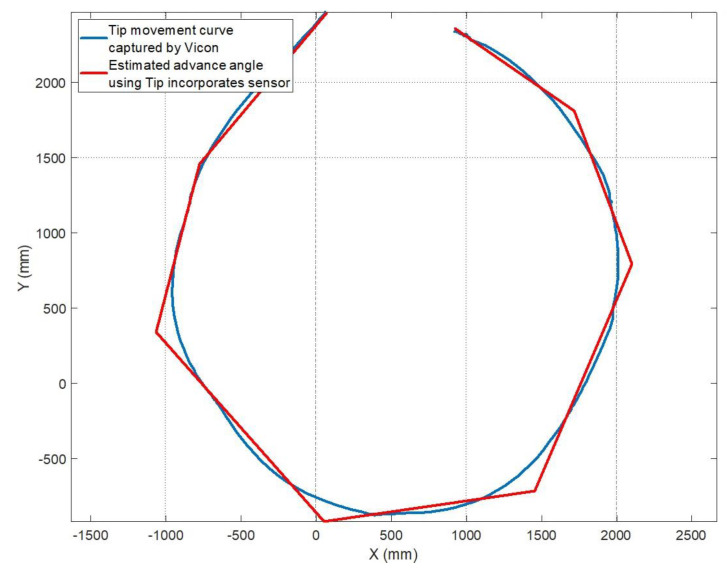
Advance plane estimation in the circular test (Worst Case Scenario) with respect to the real trajectory.

Results are summarized in Table 6, while the circular trajectory and the advance plane identification for each cycle are shown in Figure 11. As it can be seen, the proposed approach provides results with a RMS value of less than 5.6${}^{\circ }$ in the case of linear motion (45${}^{\circ }$ rotation in the handle), while the error increases up to 8${}^{\circ }$ when the trajectory is circular. This is due to the fact that the estimation plane is calculated in each step assuming that the steps are carried out linearly. Hence, this is considered a worst case scenario that reflects sharp turns while walking.

### 4.2. Anteroposterior and Lateromedial Angle Estimation

Once the body reference system $S_{B}\left(X^{\prime },Y^{\prime },Z^{\prime }\right)$ has been estimated, the anteroposterior and lateromedial angles can be calculated. As seen in the previous section,

(5) $${}^{B}R_{tip}=R_{z}\left(\theta \right)\;R_{rpy}=\left[\begin{array}{ccc} {}^{B}u_{x} & {}^{B}u_{y} & {}^{B}u_{z} \end{array}\right],$$

where ${}^{B}u_{z}={\left[\begin{array}{ccc} u_{z_{x}} & u_{z_{y}} & u_{z_{z}} \end{array}\right]}^{T}$ is the unitary directional vector of the *z* axis of the Sensorized Tip reference frame in the body reference frame. The time evolution of this vector, represents the motion of the assistive device with respect to the body reference frame.

Hence, projecting this vector in the $X^{\prime }Z^{\prime }$ and $Y^{\prime }Z^{\prime }$ planes, the anteroposterior $\theta_{ant}$ and lateromedial $\theta_{lat}$ angles can be obtained,

(6) $$\begin{array}{ccc} \theta_{ant}=atan\left(\frac{u_{z_{x}}}{u_{z_{z}}}\right)\; & \; & \theta_{lat}=atan\left(\frac{u_{z_{y}}}{u_{z_{z}}}\right). \end{array}$$

## 5. Study Case to Evaluate Device Potential with Pwms

The developed Sensorized Tip aims to monitor PwMS and provide objective and relevant data that can be used to assess patient function and progression. However, as stated in the introduction, the raw sensor and estimated orientation data defined in the previous sections should be further processed.

In this section, a set of indicators will be defined from the data provided by the Sensorized Tip (analyzed in Section 3 and Section 4). Then, the results of a exploratory study-case with PwMS will be analyzed to study the potential of the proposed device to assess patient status. Please note that this is not an exhaustive study to correlate indicators and patient status and that further thorough clinical trials are required for this latter purpose.

### 5.1. Exploratory Study-Case Setup

In order to perform the preliminary analysis, the study case was approved by the Basque Country Clinical Research Ethics Committee (CEIm) (Code PS2018017), and was carried out in collaboration with ADEMBI (Multiple Sclerosis patient Association of Biscay). This Association is exclusively dedicated to the treatment of PwMS, and provided constant supervision for the patients during the tests.

Three PwMS that required ADW, each with different functional disability status (but EDSS >4.5), volunteered for this study-case. The data of each patient is shown in Table 7, including their weight, their level of disability measured by the standarized EDSS (Expanded Disability Status Scale) [36,37] and the time to perform the TUG (Timed Up and Go) [38] standardized test.

**Figure 12:**
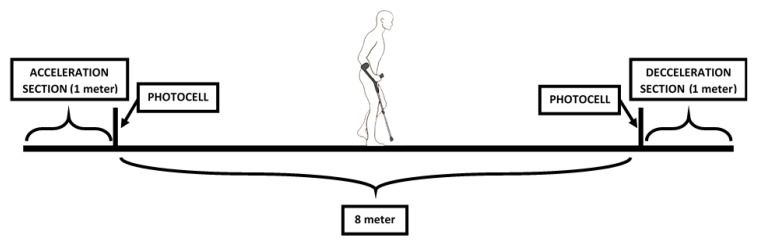
Ten meter walking test setup.

Each patient was explained the test thoroughly, and then asked to perform a standardised *10 meter walking test* (10MWT) walking at a normal and comfortable pace. The test was repeated twice, one in each direction. Note that the first meter is reserved to accelerate, while the last one to decelerate and stop. Hence, for the analysis only the middle 8 meters are considered. In order to detect these limits, two photoelectric cell gates (Polidemo, Micrgate, Italy) were placed in meters 1 and 9, allowing also to capture the average speed (see Figure 12).

### 5.2. Defined Indicators

As stated in the introduction, different indicators have been proposed in the literature based on monitoring data related to gait or ADW. In this work, some of the most relevant ones have been selected in order to demonstrate the potential of the proposed Sensorized Tip:

- The maximum load, which defines the maximum load that the user applies on the assistive device during the test (Figure 10b).
- The maximum load with respect to the weight (in percentage) [32,33], reflecting the maximum percentage of body weight the patient applies on the ADW during the stance phase.
- The average load with respect to the weight (in percentage) [33], reflecting the percentage of body weight the patient is applying on the assistive device.
- The average of two steps time [34], which represents the average time a patient requires for each cycle; this is, both the stance phase and the swing phase (Figure 10b).
- Number of cycles [34], defined by the number of cycles (stance/swing) the patient requires to complete the test.

The aforementioned indicators can be extracted from the force sensor data, as shown in Figure 10b. In addition, a set of indicators related to the orientation of the Sensorized Tip have been also defined, with the aim to define the pattern of use of the device. In this way, the anteroposterior and lateromedial angles estimated using the algorithms defined in Section 4 have been captured in three critical positions (Figure 10b): the initial contact point, the point of maximum load, and the final contact point of the stance phase.

### 5.3. Results and Discussion

Results for the three PwMS on the *10 meter walking test* are summarized in Table 8. Note that as previously noted, the aim of this work is not to carry out an exhaustive correlation analysis, but to demonstrate the potential of the use of the Sensorized Tip to assess patient functionality.

From these data several initial guidelines can be extracted. If the exerted load is considered, note that the load value can give potential information regarding patient performance only if the value is normalized with respect to the body weight. In this case, the data shows that the percentage of body weight the patient applies on the sensorized assistive device increases with the degree of disability given by the Expanded Disability Status Scale (EDSS). A similar tendency is shown if the Timed Up and Go (TUG) time is considered.

If the number of cycles is considered, note that there are significant differences between Patient 1 and 2/3. This can be related also with the anteroposterior angle amplitude, as for the same distance, a less number of steps will imply greater assistive device motions (greater anteroposterior angles).

Note however, that the number of steps, or even the step time between two steps, which may be used to estimate also the average speed, potentially do not provide significant data to assess patient status. For instance, patient 3, which has a high degree of disability, presents similar number of steps than patient 2, which has a moderate degree. Even more, if speed metrics are derived, it can be seen that patient 3 is quicker than patient 2, although his EDSS (and TUG) is higher. This is consistent with the high heterogeneity within PwMS.

On the other hand, an analysis of the assistive device motion can be carried out by analyzing the proposed indicators for the estimated anteroposterior and lateromedial angles. These data can be used to analyze the way the patient uses the device through the day, and to detect if changes have arised, as proposed by the authors. For instance, patient 1 uses the cane to take impulse, as it first contacts the ground almost vertically ($1.4^{\circ }$), moves the cane back while taking impulse (the maximum load is exerted in $7.12^{\circ }$, that is, with the cane behind the body) up to $27.4^{\circ }$. Patient 2, on the other hand, requires increased crutch support to move, starts the gait cycle by placing the crutch in front of the body ($-24^{\circ }$) and using it to take impulse while pivoting (maximum force exerted at $-14.7^{\circ }$) until reaching a near vertical position ($-5.5^{\circ }$). Finally, patient 3, starts the cycle by placing the crutch in front of the body similar to patient 2 ($-20.9^{\circ }$), but pivotes with both crutches applying increased load until reaching a near vertical position, where the maximum load is applied ($-4.36^{\circ }$), finishing the cycle almost there.

In addition to the anteroposterior angle, the lateromedial angle can also be used to complete the use-pattern of the assistive device. Note that greater lateromedial angle may be related with a greater requirement for support area. This way, patient 1 places the cane near the vertical first, and opens the angle while taking impulse. Patient 2, places the crutch with a broader angle to ensure balance prior to taking impulse, and maintains it while pivoting (note that in this case, the crutch always moves in front of the patient, as previously detailed). Finally, patient 3 places the sensorized crutch almost vertically, as the patient uses it to take impulse while pivoting.

In summary, the exerted load with respect to the body weight and the proposed orientation angles are potential indicators to be considered to analyze patient status, while parameters related to the average speed seem to have less potential for the aforementioned purpose. These guidelines demonstrate the potential of the proposed device, and define the starting point for further tests with PwMS in order to perform a full correlation analysis on the data provided by the developed device.

## 6. Conclusions

Individualized rehabilitation is mandatory for achieving the highest possible level of independence in people with Multiple Sclerosis (PwMS). For that purpose, proper monitoring devices are required, which provide objective data that allow designing patient-centered therapy.

In this work a Sensorized Tip that can be attached to any crutch or cane is proposed to monitor PwMS that require Assistive Devices for Walking (ADW) within the day. Different from other approaches, the proposed device is designed to minimize the impact on the patient and allows to collect the axial load and the ADW 3D motion data. The proposed Sensorized Tip integrates a motion processing unit that provides acceleration, rotation and inclination data, a barometer for altitude estimation and a force sensor. The device is designed to be lightweight and allows wireless communication using Bluetooth protocol.

The novel Sensorized Tip measurement errors are characterized in a series of tests, concluding that the device’s accuracy is enough to monitor PwMS. In addition, an algorithm to estimate relevant orientation data is defined. Moreover, the potential of the device to provide data to perform patient status assessments is analyzed in a exploratory case study with PwMS. Preliminary results demonstrate that the proposed device allows to extract important information regarding not only their status, but also the use given to the ADW, which has clinical repercussion.

However, the reported work presents some limitations that will be handled in future works. First, the study-case with PwMS was not carried out in 3D Motion Capture facilities due to the risks involved for the patients. Second, the weight and design of the device can be further optimized based on patient’s feedback. Finally, the proposed study case is a preliminary analysis with a reduced number of patients, and select indicators that, although demonstrates an area of research with great potential, also emphasizes the need for further research.

In this sense, future work will include working in the aforementioned limitations, being the most relevant one the need to perform a wider longitudinal study with a represenative sample of PwMS (with an N > 20 ) to analyze correlations between the parameters offered by the Sensorized Tip and validated clinical tests (EDSS, TUG test data). This analysis could allow to give more insight into the validity of the approach to assess patients, and determine if the different indicators are able to detect changes in the patient status as the disease evolves.

## Figures and Tables

**Table 1:** BLE package data.

First Package		20 Bytes	Second Package		20 Bytes
		Number of Bits			Number of Bits
Iteration		**4**	Iteration		**4**
Force		**16**	Accelerometer		**45**
Altitude		**32**		Y axis	20
Euler Angles		**78**		Z axis	25
	Roll	26	Gyroscope		**60**
	Pitch	26		X axis	20
	Yaw	26		Y axis	20
Accelerometer		**30**		Z axis	20
	X axis	25	Magnetometer		**48**
	Y axis	5		X axis	16
				Y axis	16
				Z axis	16

**Table 2:** Volunteer data for Sensorized Tip Laboratory Validation.

Volunteer	Sex	Age	Height	Weight
1	Male	24	1.84 m	75 kg
2	Male	26	1.80 m	73 kg

**Table 3:** X-Sens Euler angles estimation error.

		RMS Error		
Test	Handle Orientation	Roll (${}^{\circ }$)	Pitch (${}^{\circ }$)	Yaw (${}^{\circ }$)
Walk straight 4 meters	0°	0.5439	1.0971	2.3457
Walk straight 4 meters	45°	0.8482	0.7325	2.1725
Walk straight 4 meters	90°	0.5429	1.0984	2.3404
Zigzag	0°	0.6736	0.8693	4.3096
Circle	0°	0.935	1.5278	4.3099
Mean		0.7267	1.0777	3.4688

**Table 4:** X-Sens rotational speed measurement error summary.

	Gyroscope Error		
Motor Speed	*x* axis (${}^{\circ }/s$)	*y* axis (${}^{\circ }/s$)	*z* axis (${}^{\circ }/s$)
100°/s	–0.1463	–0.5159	–0.6127
200°/s	–0.1944	–0.9729	–1.0299
300°/s	–1.2977	–3.3778	–0.2363

**Table 5:** X-Sens acceleration measurement error summary.

	*x* (m/s${}^{2}$)	*y* (m/s${}^{2}$)	*z* (m/s${}^{2}$)
RMS Error	1.1365	0.3963	0.4574
Mean Error	0.9674	-0.3103	−0.1704

**Table 6:** Body Reference System θ offset estimation error.

Test	Handle Orientation	RMS Error	Mean Error
Walk straight 4 meters	0${}^{\circ }$	5.4556	1.7311
Walk straight 4 meters	45${}^{\circ }$	5.605	5.4886
Walk straight 4 meters	90${}^{\circ }$	2.4038	1.5265
Zigzag	0${}^{\circ }$	4.4965	–2.0318
Circle	0${}^{\circ }$	7.9843	–5.8093

**Table 7:** Selected People with Multiple Sclerosis (MS) for the exploratory study-case.

	Weight (kg)	EDSS	TUG (s)	Assistive Device for Walking
Patient 1	86.4	4.5	14.1	Cane
Patient 2	78	6.5	22.97	Crutch
Patient 3	56.8	7.5	49.29	Crutch

**Table 8:** Exploratory Study-Case results.

	Patient 1	Patient 2	Patient 3
Timed Up and Go (TUG) (s)	14.1	22.97	49.29
Expanded Disability Status Scale (EDSS)	4.5	6.5	7.5
Avgerage Speed (m/s)	0.82	0.46	0.61
Maximum Load (Kg)	6.69	10.44	11.09
Maximun Load with Respect Weight (%)	7.74	13.38	19.52
Mean Load (Kg)	5.55	7.78	6.74
Mean Load with Respect Weight (%)	6.42	9.97	11.86
Average Two Step Time (s)	1.49	1.65	1.31
Number of Cycles	6.5	10.5	10
Anteroposterior Angle Amplitude (${}^{\circ }$)	35.75	20.74	24.79
Anteroposterior Angle in Initial Support (${}^{\circ }$)	1.40	−24.00	−20.92
Anteroposterior Angle in Maximum Load (${}^{\circ }$)	7.12	−14.72	−4.36
Anteroposterior Angle in Final Support (${}^{\circ }$)	27.40	−5.52	−2.30
Lateromedial Angle Amplitude (${}^{\circ }$)	4.50	3.67	5.39
Lateromedial Angle in Initial Support (${}^{\circ }$)	6.78	10.40	6.96
Lateromedial Angle in Maximum Load (${}^{\circ }$)	8.29	9.93	4.91
Lateromedial Angle in Final Support (${}^{\circ }$)	6.17	8.74	7.60

## Abbreviations

The following abbreviations are used in this manuscript:

MS	Multiple Sclerosis
PwMS	People with Multiple Sclerosis
ADW	Assistive Devices for Walking
IMU	Inertial Measurement Unit
DAQ	Data Acquisition System
BLE	Bluetooth 4.0 Low Energy
ATT	Attribute Protocol
MCS	Motion Capturing System
RMS	Root Mean Square
EDSS	Expanded Disability Status Scale
TUG	Timed Up and Go

## References

[1] Federation M.S.I. (2008). The Atlas of Multiple Sclerosis.

[2] Organization W.H. (2006). Neurological disorders: Public health challenges.

[3] (FELEM) F.E.d.L.c.l.E.M. (2007). Esclerosis múltiple en España: Realidad, necesidades sociales y calidad de vida.

[4] Souza A., Kelleher A., Cooper R., Cooper R.A., Iezzoni L.I., Collins D.M. (2010). Multiple sclerosis and mobility-related assistive technology: Systematic review of literature. J. Rehabil. Res. Dev..

[5] Flachenecker P. (2015). Clinical Implications of Neuroplasticity – The Role of Rehabilitation in Multiple Sclerosis. Front. Neurol..

[6] Jones D.E., Armstrong M.J., Sutliff M.H., Halper J., Brown T.R., Haselkorn J.K., Kraft G.H., Narayanaswami P. (2016). Summary of comprehensive systematic review: Rehabilitation in multiple sclerosis: Report of the Guideline Development, Dissemination, and Implementation Subcommittee of the American Academy of NeurologyAuthor Response. Neurology.

[7] National Collaborating Centre for Chronic Conditions (Great Britain) and Chartered Society of Physiotherapy (Great Britain) (2004). Multiple Sclerosis: National Clinical Guideline for Diagnosis and Management in Primary and Secondary Care.

[8] Latimer-Cheung A.E., Pilutti L.A., Hicks A.L., Ginis K.A.M., Fenuta A.M., MacKibbon K.A., Motl R.W. (2013). Effects of exercise training on fitness, mobility, fatigue, and health-related quality of life among adults with multiple sclerosis: A systematic review to inform guideline development. Arch. Phys. Med. Rehabil..

[9] Shull P.B., Jirattigalachote W., Hunt M.A., Cutkosky M.R., Delp S.L. (2014). Quantified self and human movement: A review on the clinical impact of wearable sensing and feedback for gait analysis and intervention. Gait Posture..

[10] Gong J., Goldman M.D., Lach J. Deepmotion: A deep convolutional neural network on inertial body sensors for gait assessment in multiple sclerosis. Proceedings of the 2016 IEEE Wireless Health, WH 2016.

[11] Gyllensten I.C., Bonomi A.G. (2011). Identifying types of physical activity with a single accelerometer: Evaluating laboratory-trained algorithms in daily life. IEEE Trans. Biomed. Eng..

[12] Moncada-Torres A., Leuenberger K., Gonzenbach R., Luft A., Gassert R. (2014). Activity classification based on inertial and barometric pressure sensors at different anatomical locations. Physiol. Meas..

[13] Shoaib M., Bosch S., Durmaz Incel O., Scholten H., Havinga P.J. (2014). Fusion of smartphone motion sensors for physical activity recognition. Sensors.

[14] Aguiar B., Silva J., Rocha T., Carneiro S., Sousa I. Monitoring physical activity and energy expenditure with smartphones. Proceedings of the IEEE Computer Society.

[15] Brichetto G., Pedullà L., Podda J., Tacchino A. (2019). Beyond center-based testing: Understanding and improving functioning with wearable technology in MS. Mult. Scler..

[16] Sardini E., Serpelloni M., Lancini M. (2015). Wireless instrumented crutches for force and movement measurements for gait monitoring. IEEE Trans. Instrum. Meas..

[17] Culmer P.R., Brooks P.C., Strauss D.N., Ross D.H., Levesley M.C., Oconnor R.J., Bhakta B.B. (2014). An instrumented walking aid to assess and retrain gait. IEEE/ASME Trans. Mechatron..

[18] Lancini M., Serpelloni M., Pasinetti S. Instrumented crutches to measure the internal forces acting on upper limbs in powered exoskeleton users. Proceedings of the 2015 6th International Workshop on Advances in Sensors and Interfaces (IWASI).

[19] Tsuda N., Hayashi A., Tounai M., Akutagawa S. Visualization system of crutch walking based on internal sensors. In Proceeding of IEEE/ASME International Conference on Advanced Intelligent Mechatronics.

[20] Hassan M., Kadone H., Suzuki K., Sankai Y. (2014). Wearable Gait Measurement System with an Instrumented Cane for Exoskeleton Control. Sensors.

[21] Mekki F., Borghetti M., Sardini E., Serpelloni M. Wireless instrumented cane for walking monitoring in Parkinson patients. Proceedings of the 2017 IEEE International Symposium on Medical Measurements and Applications (MeMeA).

[22] Megalingam R.K., Greeshma M.G., Pillai S.S. Design and implementation of intelligent crutches for medical applications. Proceedings of the 2019 International Conference on Communication and Signal Processing (ICCSP).

[23] Seylan Ç., Saranli U. (2018). Estimation of ground reaction forces using low-cost instrumented forearm crutches. IEEE Trans. Instrum. Meas..

[24] Chamorro-Moriana G., Sevillano J., Ridao-Fernández C. (2016). A Compact forearm crutch based on force sensors for aided gait: Reliability and validity. Sensors.

[25] Merrett G.V., Ettabib M.A., Peters C., Hallett G., White N.M. (2010). Augmenting forearm crutches with wireless sensors for lower limb rehabilitation. Meas. Sci. Technol..

[26] Wade J.W., Boyles R., Flemming P., Sarkar A., De Riesthal M., Withrow T.J., Sarkar N. (2019). Feasibility of automated mobility assessment of older adults via an instrumented cane. IEEE J. Biomed. Health Inf..

[27] Chen Y.F., Napoli D., Agrawal S.K., Zanotto D. Smart crutches: Towards instrumented crutches for rehabilitation and exoskeletons-assisted walking. Proceedings of the 2018 7th IEEE International Conference on Biomedical Robotics and Biomechatronics (Biorob).

[28] Jurman D., Jankovec M., Kamnik R., Topič M. (2007). Calibration and data fusion solution for the miniature attitude and heading reference system. Sens. Actuat. A Phys..

[29] Luinge H.J., Veltink P.H. (2005). Measuring orientation of human body segments using miniature gyroscopes and accelerometers. Med. Biol. Eng. Comput..

[30] Del Rosario M., Redmond S., Lovell N. (2015). Tracking the Evolution of Smartphone Sensing for Monitoring Human Movement. Sensors.

[31] Sardini E., Serpelloni M., Lancini M., Pasinetti S. (2014). Wireless instrumented crutches for force and tilt monitoring in lower limb rehabilitation. Procedia Eng..

[32] Routson R.L., Bailey M., Pumford I., Czerniecki J.M., Aubin P.M. A smart cane with vibrotactile biofeedback improves cane loading for people with knee osteoarthritis. Proceedings of the 2016 38th Annual International Conference of the IEEE Engineering in Medicine and Biology Society (EMBC).

[33] Simic M., Bennell K.L., Hunt M.A., Wrigley T.V., Hinman R.S. (2011). Contralateral cane use and knee joint load in people with medial knee osteoarthritis: The effect of varying body weight support. Osteoarthr. Cartil..

[34] Sprint G., Cook D.J., Weeks D.L. Quantitative assessment of lower limb and cane movement with wearable inertial sensors. In Proceeding of 2016 IEEE-EMBS International Conference on Biomedical and Health Informatics (BHI).

[35] Slavens B.A., Bhagchandani N., Wang M., Smith P.A., Harris G.F. (2011). An upper extremity inverse dynamics model for pediatric Lofstrand crutch-assisted gait. J. Biomech..

[36] Kurtzke J.F. (1983). Rating neurologic impairment in multiple sclerosis: An expanded disability status scale (EDSS). Neurology.

[37] Meyer-Moock S., Feng Y.S., Maeurer M., Dippel F.W., Kohlmann T. (2014). Systematic literature review and validity evaluation of the expanded disability status scale (EDSS) and the multiple sclerosis functional composite (MSFC) in patients with multiple sclerosis. Bmc Neurol..

[38] Cattaneo D., Regola A., Meotti M. (2006). Validity of six balance disorders scales in persons with multiple sclerosis. Disabil. Rehabil..

